# 老年晚期肺癌内科治疗中国专家共识(2022版)

**DOI:** 10.3779/j.issn.1009-3419.2022.101.25

**Published:** 2022-06-20

**Authors:** 中国老年保健协会肺癌专业委员会, 北京肿瘤学会肺癌专业委员会

## 前言

肺癌是全球及我国60岁以上人群发病率及死亡率最高的恶性肿瘤^[1]^，晚期肺癌是导致死亡的主要原因。随着年龄增长，老年患者合并的基础疾病增多，同时伴有器官储备功能下降。老年患者生理机能的改变对药物的吸收、分布、代谢及清除等诸多方面都会产生影响^[2]^，并可能导致老年患者对于治疗的耐受性下降。既往研究^[3]^显示年龄65岁以上的老年患者化疗后粒细胞减少伴感染的发生率和死亡率显著升高。此外，黏膜炎、心脏及神经毒性等化疗不良反应(adverse event, AE)发生的风险和严重程度也随着年龄的增长而增加。根据美国国立癌症研究所数据库(Surveillance, Epidemiology, and End Results Program, SEER)^[4]^近40年的病例分析显示肺癌患者中老年人占比也有逐年增高的趋势。世界卫生组织(World Health Organization, WHO)对老年人的定义为60周岁以上人群^[5]^。美国国立综合癌症网络(National Comprehensive Cancer Network, NCCN)老年肿瘤指南中对于老年人的定义为65周岁以上的人群，并且这个标准自2006年一直沿用至今，进一步依据年龄把老年肿瘤患者分为如下三类：65岁-75岁为低龄老年人，76岁-85岁为老年人， > 85岁为高龄老年人^[6]^。

尽管恶性肿瘤在老年人中高发，但国内外开展的各类临床研究中纳入的老年患者比例却明显不足。美国国立癌症研究所2001年-2011年间开展的Ⅱ期-Ⅲ期临床研究^[7]^显示65岁-74岁的患者占比仅约25%，而年龄≥75岁的患者占比则不足10%，针对85岁以上的老年肿瘤患者的治疗则更少。研究^[8]^显示85岁以上的老年肺癌患者诊断时往往分期相对更晚，相同分期的预后也显著劣于85岁以下的患者，生存率随着年龄的增长而降低(*P* < 0.000, 1)。

鉴于目前针对老年晚期肺癌患者治疗的前瞻性研究数据有限，但是对老年患者治疗的关注不断增加，肿瘤内科相关领域专家共同撰写了“老年晚期肺癌内科治疗中国专家共识(2022版)”(简称“共识”)，以期为老年晚期肺癌患者的治疗提供相关循证医学证据，从而进一步推动我国老年肺癌治疗的合理化、规范化。本共识撰写是基于现有的国内外临床循证医学证据和已经发表的临床研究数据，结合专家的临床诊治经验，从临床工作中的实际问题出发进行整理撰写。主要围绕老年晚期肺癌患者的评估以及治疗方式，包括化疗、靶向治疗、抗血管生成治疗和免疫治疗等热点问题进行编写，以期能够为临床实践提供参考。

## 老年肺癌患者评估

**问题1：老年肺癌患者是否需要进行老年评估？**

**共识推荐：年龄≥65岁并且拟接受抗肿瘤治疗的肺癌患者应该进行老年评估。老年多维度评估有助于制定个体化的抗肿瘤策略，发现可干预的老年问题。**

**证据：**老年肿瘤患者的异质性大，因此更需要权衡治疗的获益与风险。单一的卡氏功能状态(Karnofsky performance status, KPS)评分或者东部肿瘤协作组体力状态评分(Eastern Cooperative Oncology Group performance status, ECOG PS)难以反映老年肿瘤患者的整体状态，用于指导治疗具有局限性。国内一项回顾性研究^[9]^纳入了120例年龄≥65岁且接受化疗的肺癌患者，结果显示不同KPS评分的患者出现≥3级化疗副反应的比例无显著差异，即单一KPS评分无法预测老年肺癌患者的化疗耐受性。国内另一项针对老年晚期非小细胞肺癌(non-small cell lung cancer, NSCLC)患者一线给予单药化疗的回顾性分析^[10]^同样显示年龄及PS评分也不能预测3级-4级的化疗AE。

由于单一的年龄、KPS评分和ECOG PS评分均无法准确指导抗肿瘤治疗，因此对于拟接受抗肿瘤治疗的老年肺癌患者需要进行老年评估，评估的内容至少应包括躯体功能状态、合并症、跌倒史、抑郁、认知及营养状态等综合内容。老年评估能发现常规肿瘤评估中疏漏的老年问题^[11, 12]^；预测年龄≥65岁老年患者的化疗风险^[13, 14]^；有助于制定个体化的抗肿瘤策略。一项包含肺癌患者的系统综述^[15]^显示经过老年评估，中位28%(范围8%-54%)的患者调整了抗肿瘤治疗方案，其中多数患者接受了更加和缓的治疗模式。此外，中位72%(范围26%-100%)的患者调整了营养及多重用药等干预。由于75%的研究显示老年评估组治疗完成率更高，55%的研究显示老年评估组AE更低，该系统综述提示老年评估可能有助于提高老年患者抗肿瘤治疗的耐受性。

首个依据老年评估制定肺癌患者治疗方案的前瞻性随机对照研究发表于2016年^[16]^，研究纳入了494例年龄≥70岁的晚期NSCLC患者。研究组依据老年评估的结果制定治疗策略(健康组采用含铂双药化疗，脆弱组采用单药化疗，而衰弱组仅采用姑息治疗)，对照组则根据年龄和PS评分制定化疗方案(PS≤1分且年龄≤75岁者采用含铂双药化疗，而PS评分2分或者年龄 > 75岁者采用单药化疗)。结果显示老年评估组中位总生存时间(median overall survival, mOS)(6.1个月 *vs* 6.4个月，*P*=0.87)与对照组无显著差异，但老年评估组所有级别AE的发生率显著低于常规治疗组(85.5% *vs* 93.4%, *P*=0.01)，且有接近1/4的患者避免了接受化疗。

老年评估对于老年晚期肺癌患者靶向治疗及免疫治疗的指导意义目前尚在研究探索之中。近期一项包含肺癌的前瞻性队列研究^[17]^提示采用老年筛查工具-8(Geriatric-8, G-8)量表评分 < 15分的患者不仅死亡风险更高(*P*=0.01)，免疫治疗后因不良事件再入院治疗的比例也更高(*P*=0.031)。该研究提示老年评估可能有助于甄别免疫治疗后容易出现严重不良事件的高危脆弱老年患者。因此，抗肿瘤治疗前先进行老年评估，依据评估结果给予个体化的治疗方案，可以更好地保证治疗的安全性，在精准治疗时代，老年评估对于老年肺癌患者的意义尤为重要。

**问题2：哪些工具可用于评估老年肺癌患者的化疗耐受性？(附量表)**

**共识推荐：癌症及衰老研究组(cancer aging research group, CARG)化疗风险评估量表及老年化疗风险评估量表(chemotherapy risk assessment scale for high-age patients, CRASH)等量表可用于预测老年肺癌患者的化疗耐受性。**

**证据：**目前推荐可用于老年评估的工具包括：采用工具性日常生活活动能力量表(instrumental activities of daily living, IADL)及日常生活能力量表(activities of daily living, ADL)评估功能，采用Charlson合并症指数(Charlson comorbidity index, CCI)或老年累计疾病评分表(cumulative illness rating scale-Geriatric, CIRS-G)评估合并症，采用简易精神状态检查表(mini-mental state examination, MMSE)评估认知状态，采用老年抑郁量表(geriatric depression scale, GDS)筛查抑郁，询问是否存在跌倒等老年综合征，以及通过体重变化等评估营养状态^[18]^。此外，G-8还有助于评估老年肺癌患者的死亡风险^[19]^。

回顾性研究显示老年患者同样能从化疗中获益，但AE的发生率较年轻人升高，因此在化疗前需要进行化疗风险评估。CARG化疗风险评估量表临床可操作性较强，可用于治疗前评估化疗风险，有助于制定老年肺癌患者个体化的治疗方案。量表内容包括年龄等患者基本信息、肿瘤类型、化疗方案、血清指标及简易老年评估。一项针对年龄≥65岁、样本量为500例且包含29%肺癌患者的前瞻性队列研究^[13]^显示CARG化疗风险评估量表能预测老年患者的化疗耐受性。量表评估为低危、中危及高危的患者出现3级-5级化疗副反应的比例分别为30%、52%和83%(*P* < 0.001)，有显著差异。此外，还有两个类似的研究^[9, 14]^也再次验证了该研究结论。CRASH量表则是另一项较为全面的老年评估工具，涵盖了功能、认知、营养等维度的评估，测评通常需要20-30分钟完成。一项国外的前瞻性队列研究^[20]^，共入组562例患者，518例可评价患者中有20%为肺癌，结果显示CRASH量表可以分别预测血液学毒性和非血液学毒性的发生率，验证了CRASH量表对于年龄≥70岁的老年肿瘤患者化疗耐受性的预测能力。

国内外多项研究^[21, 22]^显示无论CARG化疗风险评估量表还是CRASH量表均能预测老年肿瘤患者的化疗副反应，且二者的预测效能类似。此外，也有研究^[9, 23]^提示合并症及老年脆弱性问卷(Vulnerable Elders Survey-13, VES-13)等评估也有助于判断老年肺癌患者的化疗耐受性。

**问题3：老年肺癌患者药物治疗的注意事项？**

**共识推荐：老年肺癌患者普遍存在多重用药，治疗过程中应注意药物间的相互作用及患者的肝肾功能，依据个体情况调整抗肿瘤药物的剂量。**

**证据：**老年肿瘤患者因合并多种疾病，因此常见抗肿瘤治疗同时伴有相关疾病的多重用药。研究^[24]^显示老年肿瘤患者中位合并用药数多达5个-9个。回顾性研究^[25]^显示35%的肿瘤患者存在严重的药物相互作用。多因素分析显示≥5个合并用药，住院患者以及肺癌患者出现严重药物相互作用的风险会增加。治疗过程中应注意判断是否存在药物间的相互作用，药物与疾病的相互作用以及药物AE，例如：卡铂、依托泊苷、吉西他滨、紫杉醇和吉非替尼可增加华法林的血药浓度，导致出血风险增加；顺铂可降低苯妥英钠的血药浓度，因而不利于癫痫的控制；一代和三代表皮生长因子受体(epidermal growth factor receptor, EGFR)-酪氨酸激酶抑制剂(tyrosine kinase inhibitors, TKIs)主要经CYP3A4/5代谢，卡马西平及苯妥英钠等强CYP酶诱导剂能加速一代和三代EGFR-TKIs代谢，降低其血药浓度，继而会影响抗肿瘤疗效；伊曲康唑等强CYP抑制剂能增加一代EGFR-TKIs的血药浓度，而导致药物AE增加；胃液pH值升高会减少一代EGFR-TKIs的吸收等。基于SEER数据库的研究显示对于≥65岁的老年肿瘤患者，TKIs治疗期间联合应用质子泵抑制剂(proton pump inhibitors, PPIs)的比例高达22%。厄洛替尼联合PPIs治疗的肺癌患者90 d内死亡风险增加21%，1年内死亡风险增加11%^[26]^。吉非替尼的研究^[27]^同样也提示联合PPIs治疗会增加患者的死亡风险。此外，有研究^[28]^提示PPIs还可能影响免疫检查点抑制剂治疗的疗效。POPLAR及OAK研究的汇总分析提示免疫检查点抑制剂联合PPIs治疗的患者无论无进展生存时间(progression free survival, PFS)(1.9个月 *vs* 2.8个月，*P*=0.001)还是OS(9.6个月 *vs* 14.5个月，*P*=0.000, 1)均显著缩短。

老年肺癌患者合并肝肾功能不全的比例增高，故对于经肝肾代谢的抗肿瘤药物需要调整剂量以降低其毒副反应。需要依据肾功能调整剂量的药物包括：顺铂、卡铂、培美曲塞、依托泊苷、克唑替尼等。轻、中度肝功能不全需要调整的药物包括：多西他赛、紫杉醇、白蛋白紫杉醇、吉西他滨、吉非替尼、厄洛替尼、克唑替尼、布加替尼等。重度肝功能不全需要调整剂量的药物包括：阿来替尼、塞瑞替尼、奥希替尼、培美曲塞、依托泊苷、长春瑞滨等^[29]^。

因此对于老年肺癌患者在治疗过程中要了解合并用药情况并注意监测用药过程中肝肾功能情况，必要时给予治疗药物和剂量的调整。

## 老年晚期NSCLC的化疗选择

**问题1：老年晚期NSCLC患者接受化疗是否能够获得临床获益？**

**共识推荐：老年晚期NSCLC患者接受化疗有临床获益，对于可以耐受化疗的老年患者，化疗优于最佳支持治疗。**

**证据：**ELVIS研究^[30]^是一项在意大利开展的多中心、前瞻性Ⅲ期临床研究，共纳入了191例70岁以上ECOG PS评分为0分-2分的老年晚期NSCLC患者，随机分为单独接受最佳支持治疗组(best supportive care, BSC)和长春瑞滨联合BSC组。研究结果显示相较于BSC组，化疗组可以显著延长中位生存期(mediansurvivaltime, MST)(28周 *vs* 21周，*P*=0.03)，提高1年生存率(32% *vs* 14%)，改善生活质量(quality of life, QOL)。化疗组整体耐受性良好，10%的患者出现3级/4级中性粒细胞减少，16%的患者出现2级/3级贫血。其后的一项回顾性研究^[31]^也证实了ELVIS研究的结果，该研究对SEER数据库中21, 285例年龄 > 65岁的晚期NSCLC患者数据进行分析，结果显示，接受化疗相较于BSC能显著降低死亡风险(HR=0.77, *P* < 0.000, 1)。另一项Ⅲ期研究^[32]^的亚组分析显示，对于PS评分为0分或1分的患者，≥70岁患者一线接受吉西他滨或紫杉醇联合卡铂或吉西他滨联合紫杉醇三种不同的化疗方案治疗后的生存获益及安全性与 < 70岁患者相似。以上研究提示，PS评分状况较好的老年晚期NSCLC患者接受化疗可有临床获益。

此外，一项2005年发表的主要针对老年晚期NSCLC患者最佳治疗选择的研究^[33]^，共有来自欧洲5个国家的12位肿瘤专家根据已经发表的研究数据结果进行汇总分析后，建议老年晚期NSCLC患者选择第三代药物化疗；对于器官功能良好的老年患者可以选择含铂方案化疗，对于不适合接受抗肿瘤治疗的老年晚期NSCLC患者可选择最佳支持治疗。但目前研究数据多是基于欧美人群的研究，对于我国老年肺癌人群，尤其是高龄老年患者的化疗是否获益，尚无明确研究证据。

**问题2：老年晚期NSCLC患者常用的化疗药物及治疗方案的选择？**

**共识推荐：老年晚期NSCLC患者接受含铂双药治疗优于单药化疗，但需考虑患者身体状况选择不同的化疗药物及治疗方案并注意AE。**

**证据：**多项临床研究证实第三代化疗药物如长春瑞滨、吉西他滨、紫杉醇、多西他赛、培美曲塞等能改善老年晚期NSCLC患者的生存和提高QOL。来自意大利的ELVIS研究^[30]^证实了长春瑞滨单药对老年晚期NSCLC患者的有效性和安全性。随后MOVE等研究^[34, 35]^结果显示，对于不能耐受含铂双药化疗的老年晚期NSCLC患者，口服长春瑞滨节拍化疗是有效且安全的治疗选择。吉西他滨也是治疗NSCLC常用药物之一，多项前瞻性Ⅱ期临床研究^[36-40]^显示吉西他滨单药在 > 70岁的老年晚期NSCLC患者中具有良好的疗效，MST约为6.8个月-9个月，客观缓解率(objective response rate, ORR)达18%-38%，Ⅳ级血液学毒性如白细胞、中性粒细胞、血小板减少或贫血的发生率通常低于5%。紫杉醇类药物(紫杉醇^[41-43]^和多西他塞^[44, 45]^)在治疗老年晚期NSCLC中MST为7.8个月-12个月，ORR为23%-44%。一项Ⅱ期研究^[46]^中纳入46例75岁以上老年非鳞癌NSCLC患者接受培美曲塞单药一线治疗，中位无进展生存时间(median progression free survival, mPFS)为4.9个月，ORR为13%，mOS为18.2个月，且耐受性良好。

法国肺癌协作组进行的大型Ⅲ期随机对照IFCT 0501研究^[47]^，比较了紫杉醇和卡铂联合方案与单药(长春瑞滨或吉西他滨)治疗老年晚期NSCLC的有效性和安全性，研究共纳入451例 > 70岁的老年晚期NSCLC患者，中位年龄77岁，结果显示mPFS 6.0个月 *vs* 2.8个月(*P* < 0.001)；mOS 10.3个月 *vs* 6.2个月(*P* < 0.000, 1)联合组显著优于单药组，疾病控制率(disease control rate, DCR)也更高(65.3% *vs* 56.2%, *P*=0.047)。联合治疗组化疗相关毒性的发生率亦明显增加，最常见的是中性粒细胞计数减少(48.4% *vs* 12.4%)和乏力(10.3% *vs* 5.8%)。一项对比培美曲塞联合卡铂和培美曲塞单药治疗PS评分为2分的晚期非鳞状NSCLC患者的疗效的Ⅲ期研究^[48]^的亚组分析显示，对于老年NSCLC患者，接受培美曲塞联合卡铂较培美曲塞单药可以延长OS。之后日本的一项多中心、随机对照的Ⅲ期研究^[49]^比较了培美曲塞联合卡铂对比多西他赛一线治疗75岁以上的晚期NSCLC患者，研究结果显示培美曲塞联合卡铂组的PFS和OS均显著优于多西他赛单药组，两组的AE均可耐受。另一项荟萃分析^[50]^纳入10项研究共2, 510例患者，比较第三代化疗药物单药治疗和联合铂类双药化疗作为 > 65岁老年晚期NSCLC患者一线治疗的疗效，结果显示含铂双药化疗组的PFS、OS、ORR、1年生存率均优于单药治疗，但3级/4级贫血、血小板减少和神经毒性等AE的发生率也更高。因此，对于老年晚期NSCLC含铂双药方案较单药显示出生存获益，但是AE发生率也会有所增加。

由于第三代化疗药物单药治疗老年晚期NSCLC患者的有效性和安全性均良好，非铂类的第三代药物双药联合治疗也受到关注。大型Ⅲ期随机对照MILE研究^[51]^入组了698例70岁以上的晚期NSCLC患者，随机分配接受长春瑞滨、吉西他滨和长春瑞滨联合吉西他滨三个不同治疗组，结果显示与单药治疗相比，联合治疗并没有显著改善患者生存，虽然三个治疗组的QOL相似，但联合治疗相较于单药治疗毒性反应更大。此外，其他几项研究，如吉西他滨单药对比多西他塞联合吉西他滨^[52, 53]^、培美曲塞单药对比培美曲塞联合吉西他滨^[54]^治疗也得出了类似结果。因此根据上述研究结果显示对于老年晚期NSCLC非铂类的第三代药物双药联合治疗的临床获益并不优于单药治疗。

综上，建议对老年晚期NSCLC患者根据身体状况选择含铂双药或是单药进行化疗，身体状况不允许化疗的患者则给予对症支持治疗。

## 老年晚期NSCLC抗血管生成治疗

**问题1：老年NSCLC患者抗血管生成治疗药物的应用及安全性如何？**

**共识推荐：老年NSCLC患者可考虑采用与总体人群相似治疗剂量，安全性整体亦大致相似。但在应用抗血管生成药物期间需要对AE进行严密监测。**

**证据：**贝伐珠单抗是临床常用的人源化血管内皮生长因子(vascular endothelial growth factor, VEGF)单克隆抗体，常规推荐剂量为15 mg/kg/次，每3周1次。ECOG 4599研究^[55]^亚组分析显示，相较于年龄 < 70岁的肺癌患者，贝伐珠单抗15 mg/kg联合化疗在年龄≥70岁的老年患者中，3级及以上治疗相关不良事件发生率显著升高(87% *vs* 70%, *P* < 0.001)。但随后的AVAiL^[56]^、SAiL^[57]^、ARIES^[58]^等研究表明，不同剂量的贝伐珠单抗(7.5 mg/kg或15 mg/kg)联合化疗的AE多在2级以内，3级及以上不良事件发生率在老年患者(年龄≥65岁或≥70岁)和非老年患者中未见统计学差异，且未观察到老年患者组贝伐珠单抗治疗相关毒性发生率的增加。总体来说，无论以65岁、70岁或75岁作为老年患者年龄的分界值，不同剂量贝伐珠单抗联合化疗在老年肺癌患者中的耐受性与非老年患者相当。因此可采用与总体人群类似的贝伐珠单抗推荐剂量。但由于老年患者常合并高血压、冠状动脉粥样硬化性心脏病等心脑血管基础疾病，AE发生的风险会有所升高，因此需针对贝伐珠单抗使用过程中可能出现的AE进行更加严密的监测。

REVEL研究^[59]^中，雷莫芦单抗10 mg/kg联合多西他赛治疗NSCLC患者的研究中，中位年龄为62岁，其中38%为年龄≥65岁的患者。该研究虽未单独分析老年患者对雷莫芦单抗治疗的耐受性，但在涉及其他实体瘤包括胃癌、乳腺癌的Ⅲ期研究中，雷莫芦单抗单药或联合治疗并未显示出与年龄相关的安全性差异^[60-62]^。2014年10月美国食品药品监督管理局(Food and Drug Administration, FDA)批准雷莫芦单抗联合多西他赛用于转移性NSCLC的二线治疗，推荐应用剂量为10 mg/kg，该药物尚未在中国获批适应证治疗晚期NSCLC。此外，年龄≥60岁的老年患者接受重组人血管内皮抑制素7.5 mg/m2剂量联合化疗，在药物安全性上未观察到与非老年患者间存在差异^[63]^。

抗血管小分子TKI安罗替尼在ALTER0303研究中共纳入了28例年龄≥70岁的老年NSCLC患者，其中安罗替尼试验组患者平均年龄为72岁，安全性分析提示安罗替尼常规推荐剂量在老年NSCLC患者中总体耐受性良好，未进行过剂量下调，最常见AE为高血压(81.25%)、手足综合征(75%)及血促甲状腺激素升高(68.75%)^[64]^。阿帕替尼的研究^[65]^提示，每日250 mg单药口服作为二线/三线治疗既往化疗失败的NSCLC患者，未在60岁以上老年患者和60岁以下患者中观察到药物安全性差异，AE均为2级及以下。目前，针对抗血管小分子TKI治疗的研究均为小样本的亚组数据分析，尚需进一步积累更多临床数据。

因此，在贝伐珠单抗、雷莫芦单抗及安罗替尼的临床研究中均纳入了部分老年NSCLC患者，采用了与整体人群相似的治疗剂量，耐受性与非老年患者相似。

**问题2：抗血管生成治疗药物在老年晚期肺癌患者中的治疗疗效如何？**

**共识推荐：抗血管生成治疗药物单药使用或联合化疗、EGFR-TKIs或免疫检查点抑制剂等均在老年患者中显示出了一定的疗效，在老年肺癌患者中可根据病情及身体状况酌情应用。**

**证据：**目前抗血管生成药物单药治疗晚期NSCLC的研究主要以VEGFR为主要靶点的小分子多靶点TKIs为主，如安罗替尼和阿帕替尼等。ALTER0303研究^[64]^中，安罗替尼对比安慰剂治疗年龄≥60岁或≥70岁的老年患者，在PFS和OS方面较安慰剂组均显示出明显的生存获益，尤其在≥70岁的老年患者中生存获益更加明显(OS: HR=0.34, PFS: HR=0.22)。在阿帕替尼二线/三线单药治疗标准化疗失败的NSCLC，亦未观察到年龄≥60岁和 < 60岁患者在PFS方面存在疗效差异(mPFS：3.2个月 *vs* 4.0个月，*P*=0.203)^[65]^。

在抗血管生成药物联合化疗方面，ECOG 4599研究亚组分析和PointBreak研究^[55, 66]^结果表明，联合治疗组未在年龄≥70岁或者≥75岁的老年患者中获得生存获益。但随后的AVAiL研究^[56]^表明，贝伐珠单抗联合化疗相对于化疗，在65岁以上的老年患者中看到了PFS和ORR提高。同样SAiL研究^[57]^也表明贝伐珠单抗联合化疗在老年患者和非老年患者两组的ORR和DCR基本相似，mOS均为14.6个月。同样在70岁以上的老年患者和70岁以下患者中，亦观察到相似结果。此外，ARIES研究^[58]^也证实一线贝伐珠单抗联合化疗无论在≥65岁还是≥75岁的老年患者中，相对于非老年患者，mPFS及mOS均类似，研究未发现PFS因不同年龄分组而存在差异。但上述研究在≥65岁或≥75岁的老年患者中均未见到PFS转化为OS获益，这与ECOG4599和PointBreak研究中观察到的结果一致。此外，针对晚期复发或转移性老年非鳞NSCLC患者(年龄 > 65岁)一线接受培美曲塞联合贝伐珠单抗7.5 mg/kg对比培美曲塞和卡铂联合贝伐珠单抗7.5 mg/kg治疗的非劣效性Ⅲ期随机对照研究(65Plus)^[67]^结果表明， > 70岁的老年患者接受培美曲塞和卡铂联合贝伐珠单抗的生存获益相对培美曲塞单药联合贝伐珠单抗不明显；70岁以下老年患者一线接受培美曲塞和卡铂联合贝伐珠单抗治疗的生存获益明显优于培美曲塞单药联合贝伐珠单抗(mPFS：8.2个月 *vs* 4.9个月，*P*=0.0271；mOS：17.3个月 *vs* 9.7个月，*P*=0.003, 9)。此外，该研究发现一般状态良好(PS 0分-1分)的老年患者一线接受培美曲塞和卡铂联合贝伐珠单抗治疗的生存获益也明显优于培美曲塞单药联合贝伐珠单抗(mPFS：6.9个月 *vs* 5.1个月，HR=1.353，*P*=0.029)；而对于一般状态较差(PS 2分)的老年患者，从培美曲塞单药联合贝伐珠单抗治疗中获益更多。

在抗血管生成药物联合小分子TKI方面，NEJ026研究^[68]^中，无论 < 75岁的*EGFR*突变NSCLC患者，还是≥75岁的老年患者，均能从厄洛替尼联合贝伐珠单抗治疗中得到PFS获益(HR: 0.61 *vs* 0.54)。JO25567研究^[69]^中厄洛替尼联合贝伐珠单抗在年龄≥75岁的老年患者中PFS获益趋势更高，HR低于75岁以下患者(HR: 0.23 *vs* 0.60)。RELAY研究^[70]^亚组分析也表明，相对于安慰剂联合厄洛替尼，≥65岁的老年患者接受雷莫芦单抗联合厄洛替尼治疗*EGFR*突变型NSCLC，PFS明显获益(19.4个月 *vs* 12.5个月，*P*=0.000, 9)。

抗血管生成药物联合免疫检查点抑制剂在老年肺癌患者中的疗效数据报道较少。回顾性研究^[71]^数据显示，帕博利珠单抗联合安罗替尼对比帕博利珠单抗单药治疗既往治疗失败的*EGFR*突变NSCLC，在mPFS(3.24个月 *vs* 1.50个月，*P*=0.002) 及mOS(14.92个月 *vs* 7.41个月，*P*=0.019)上均明显获益，且帕博利珠单抗联合安罗替尼治疗未在 > 65岁老年患者和非老年患者中见到PFS及OS的统计学差异。

综上，抗血管生成治疗药物联合化疗、EGFR-TKI或免疫检查点抑制剂等均在老年患者中显示出了一定的疗效，小分子TKI单药在老年患者中也有临床获益。

## 老年晚期NSCLC靶向治疗

**问题1：老年NSCLC患者驱动基因谱是否有别于其他年龄段患者？**

**共识推荐：NSCLC老年患者驱动基因谱有其特点，但与非老年患者差异不显著。对于老年肺腺癌或是有腺癌成分的患者，应常规进行驱动基因检测；部分鳞癌患者，也可以考虑进行驱动基因检测。**

**证据：**针对携带驱动基因的老年NSCLC患者，多项研究^[72-76]^表明，接受针对相应驱动基因的特异性靶向药物治疗，可以改善患者的预后、QOL，延长患者生存。靶向药物副作用相对小，耐受性较好，推荐老年晚期NSCLC患者进行驱动基因检测，靶向药物治疗是老年晚期NSCLC驱动基因阳性患者系统性治疗的首选。

目前针对老年肺癌患者的驱动基因谱的研究尚缺乏。有多个研究开展了中国NSCLC患者驱动基因图谱分析，部分研究根据患者年龄进行分析，但是各种年龄划分相对差异较大。中国NSCLC人群驱动基因分析结果显示，老年患者*EGFR*突变发生率与整体人群差异性不大，*EGFR*突变率在不同年龄段无明确的年龄相关性差异，在整体NSCLC人群*EGFR*突变发生率在39.2%-59.4%^[77-79]^，其中腺癌患者*EGFR*突变发生比例更高；*ALK*融合基因在老年患者中的发生率低于年轻患者，为3.1%-5.9%^[77-79]^；*ROS1*融合基因阳性患者较少见于60岁以上的肺癌患者，中位年龄56岁-59岁^[80-82]^。*MET*外显子14跳跃突变在老年NSCLC患者中整体发生率约1.9%^[83-85]^，高于年轻患者，在腺癌及肉瘤样癌发生率更高；针对*MET*基因治疗的相关临床研究提示入组患者中位年龄为69岁-74岁。其他的基因异常，例如*KRAS*突变、*RET*融合基因检测、*BRAF*突变、*HER*-2基因异常等，在老年NSCLC患者均有一定比例的检出率，但与年轻患者差别不大^[78, 86]^。综上，NSCLC老年患者驱动基因谱具有一定特点，但与年轻患者差异不显著。

对于老年NSCLC患者，所有含腺癌成分的NSCLC患者，应常规进行*EGFR*突变、*ALK*及*ROS1*融合基因检测，在有足够肿瘤组织标本的情况下，建议同时进行*RET*融合基因、*KRAS*突变、*BRAF*基因V600E突变、*HER-2*基因突变、*NTRK*融合基因、*MET*基因扩增及*MET*基因外显子14跳跃突变等驱动基因的检测。对于老年鳞癌患者尤其是不吸烟且为小标本活检确诊者，也可以考虑进行上述分子检测。在部分老年患者，如果难以获取足够的肿瘤组织样本进行驱动基因检测时，可采用外周血游离肿瘤DNA(cell-free/circulating tumor DNA, cf/ctDNA)进行基因检测^[87, 88]^，以指导进一步的靶向药物治疗。

综上，NSCLC老年患者驱动基因谱具有一定特点，但与非老年患者差异不显著。对于老年肺腺癌或是有腺癌成分的患者，应常规进行驱动基因检测；部分鳞癌患者，也可以考虑进行驱动基因检测。

**问题2：*EGFR*敏感突变的老年晚期NSCLC患者治疗方案如何选择？高龄患者是否从EGFR-TKIs中获益？**

**共识推荐：*EGFR*敏感突变的晚期NSCLC老年患者，一线推荐EGFR-TKIs治疗，与总体人群具有相似的生存获益，即使高龄患者也可以从EGFR-TKIs中获益。**

**证据：**目前在中国获批上市的EGFR-TKIs包括第一代EGFR-TKIs如：吉非替尼、厄洛替尼、埃克替尼；第二代EGFR-TKIs如阿法替尼、达克替尼；以及第三代如奥希替尼、阿美替尼和伏美替尼等。针对*EGFR*敏感突变NSCLC患者的一线治疗，吉非替尼、厄洛替尼、埃克替尼、阿法替尼、达克替尼、奥希替尼、阿美替尼在我国均已获批一线治疗适应证。IPASS研究^[72]^、OPTIMAL研究^[89]^、EUROTAC研究^[90]^和LUX-Lung 6临床研究^[91]^中，吉非替尼、厄洛替尼、阿法替尼对比化疗一线治疗*EGFR*突变晚期NSCLC研究，均进行了年龄相关分层分析，结果提示年龄≥65岁患者与 < 65岁患者疗效相似，吉非替尼、厄洛替尼和阿法替尼与化疗比较可显著延长患者PFS。ARCHER1050研究中^[92, 93]^，达克替尼对比吉非替尼，年龄亚组分析结果显示，年龄≥65岁患者应用达可替尼可显著延长PFS(HR=0.69, 95%CI: 0.48-0.99)，但是OS获益不显著(HR=0.987, 95%CI: 0.687-1.419)。FLAURA研究^[73, 94]^中，奥希替尼对比一代EGFR-TKIs年龄相关亚组分析结果显示，年龄≥65岁患者应用奥希替尼可显著延长PFS(HR=0.49, 95%CI: 0.35-0.67)，但是OS获益不显著(HR=0.87, 95%CI: 0.63-1.22)。综上所述，年龄≥65岁*EGFR*敏感突变的NSCLC患者，一线治疗可选择一代、二代或三代EGFR-TKIs治疗。对于一代EGFR-TKIs耐药后伴有*EGFR* 20外显子T790M突变的患者，三代EGFR-TKIs奥希替尼^[95]^、阿美替尼或伏美替尼^[96]^可作为治疗选择，年龄相关亚组分析显示，年龄≥65岁患者与 < 65岁患者相似，均可以从第三代EGFR-TKIs中获益。

针对老龄患者以及高龄患者，不同EGFR-TKIs药物开展了相应的临床研究。NEJ001、NEJ002、NEJ003汇总研究^[97]^显示：年龄≥70岁患者应用吉非替尼mPFS为14.3个月，较化疗组mPFS 5.7个月显著延长(*P* < 0.001)。针对老年患者应用厄洛替尼的研究^[98]^显示，年龄≥75岁患者相比 < 75岁患者，生存获益无显著差异，且副作用无差异。我国单中心研究^[99]^显示，≥75岁患者一线应用埃克替尼mPFS为(17.9±9.9)个月；一项中国多中心埃克替尼Ⅳ期研究^[100]^显示，年龄≥70岁患者的药物AE与总人群类似。另一项研究^[101]^显示年龄≥70岁患者应用阿法替尼mPFS为12.9个月，但40例患者中有19例患者治疗过程中需要减量，8例患者因AE停药。在年龄≥75岁伴有*EGFR*外显子20 T790M突变阳性的NSCLC患者应用奥希替尼治疗研究中^[102]^，mPFS为11.9个月(95%CI: 7.9-17.5)，mOS为22.0个月(95%CI：16.0-未达到)。针对超高龄患者，一项针对年龄≥80岁*EGFR*突变阳性NSCLC患者接受EGFR-TKIs治疗(54.4%吉非替尼，39.6%厄洛替尼，1.8%阿法替尼，4.2%未知)的多中心真实世界研究^[103]^显示，mPFS为11.9个月(95%CI：8.6-14.7)，mOS达20.9个月(95%CI: 14.3-27.1)，而且AE可耐受。上述研究显示，老龄及高龄患者均可从EGFR-TKIs中获益，且药物耐受性好。

综上，对于*EGFR*敏感突变的晚期NSCLC老年患者，一线推荐EGFR-TKIs治疗。*EGFR*敏感突变的老年晚期NSCLC患者与总体人群具有相似的生存获益，即使高龄患者也可以从EGFR-TKIs中获益。

**问题3：非*EGFR*驱动基因阳性老年晚期NSCLC患者治疗方案选择？**

**共识推荐：针对*ALK*融合基因阳性及其他少见驱动基因阳性的老年NSCLC患者，推荐给予针对性的靶向药物治疗。**

**证据：**目前国内获批的治疗*ALK*融合基因阳性晚期NSCLC药物包括克唑替尼、阿来替尼、塞瑞替尼和恩沙替尼。目前尚无针对*ALK*融合基因阳性的老年患者开展的前瞻性临床研究，在大多数ALK-TKIs的临床试验中老年患者所占比例较低，占10%-20%。PROFILE1014研究^[75]^的亚组数据结果显示，对于年龄≥65岁的老年患者，克唑替尼相比化疗PFS获益不明确(HR=0.90, 95%CI: 0.43-1.87)。在ASCEND-4研究中^[104]^，年龄≥65岁患者应用塞瑞替尼相比化疗可显著延长PFS(HR=0.45, 95%CI: 0.24-0.86)。在ALEX研究^[74]^中，年龄≥65岁患者应用阿来替尼相比克唑替尼治疗可显著延长PFS(HR=0.45, 95%CI: 0.24-0.87)。

此外，针对ALK-TKIs治疗老年NSCLC患者的回顾性分析和真实世界研究亦有报道，在一项以色列的老年肺癌患者接受ALK-TKIs(克唑替尼、塞瑞替尼、阿来替尼)治疗的真实世界回顾性分析^[105]^中，年龄 < 65岁(*n*=34)和≥65岁(*n*=19)的患者，未观察到两组患者在应用不同ALK-TKIs治疗后PFS上的显著差异，虽然在数值上 < 65岁患者OS数据更优，但受样本量限制，年龄因素对患者OS获益的影响并不具有显著性，整体上，≥65岁患者更容易产生不同程度的相关AE，其中腹泻、恶心、肌酐升高和液体潴留的表现最为明显；3级-5级AE以及因AE导致治疗停止的发生率在≥65岁组患者更高。对于老年患者，阿来替尼的安全性相对更优。另一项来自日本的回顾性分析^[106]^显示无论以65岁或者70岁作为分界点，老龄与非老龄NSCLC患者接受ALK-TKIs治疗的PFS和OS均无显著差异，而对于≥65岁患者，女性、PS评分0分、不吸烟及手术治疗都是预后良好的因素，而年龄对于预后影响并不显著。综合这两项*ALK*融合基因阳性NSCLC真实世界研究^[105, 106]^，年龄≥65岁患者与 < 65岁患者相比较，应用ALK-TKIs治疗的疗效和预后无显著差异。因此，阿来替尼可以作为*ALK*融合基因阳性的老年NSCLC患者的一线治疗药物，克唑替尼、恩沙替尼、塞瑞替尼也是可选择的治疗药物。

*MET* 14外显子跳跃突变多发生于在年龄≥70岁老年NSCLC患者^[84, 85]^，目前赛沃替尼是国内唯一获批适应证的针对该基因的靶向药物，用于含铂化疗后疾病进展或不耐受标准含铂化疗的*MET*外显子14跳跃突变的患者，其注册研究入组了70例*MET*外显子14跳跃突变的中国患者，有效率达49.2%，DCR为93.4%，mPFS达6.8个月，mOS尚未达到^[107]^。针对*MET*外显子14跳跃突变的靶向药物还有美国获批的卡马替尼(Capmatinib, INC280)和日本获批的特波替尼(Tepotinib)，但目前尚没有国内人群的研究数据和临床适应证。

此外，对于NSCLC其他少见靶点，由于发病率低，专门针对老年患者开展的研究更少，由于靶向治疗的疗效及安全性均优于化疗，因此对于驱动基因阳性的老年患者仍推荐靶向治疗作为首选，如：*ROS1*融合基因患者推荐克唑替尼^[76]^或恩曲替尼(后者我国尚未获批适应证)^[108]^治疗；*BRAF* V600E患者推荐达拉非尼联合曲美替尼治疗^[109]^；*RET*融合基因的患者可选择普拉替尼^[110]^或Selpercatinib(后者我国尚未获批适应证)^[111]^治疗；*NTRK*融合基因患者给予恩曲替尼(我国尚未获批适应证)^[112]^或拉罗替尼(我国尚未获批适应证)^[113]^治疗。

综上，*ALK*融合基因阳性老年晚期NSCLC患者，可首选ALK-TKIs治疗；*MET*外显子14跳跃突变可选择赛沃替尼作为靶向药物治疗；针对其他少见的驱动基因阳性患者，推荐选择针对性的靶向药物治疗。

## 老年晚期NSCLC免疫检查点抑制剂(immune checkpoint inhibitors, ICIs)治疗

**问题1：免疫检查点抑制剂单药治疗老年晚期NSCLC的疗效如何？**

**共识推荐：细胞程序性死亡-配体1(programmed cell death ligand 1, PD-L1)高表达老年晚期NSCLC一线推荐ICIs单药治疗，二线及以上治疗ICIs与化疗相比能带来生存获益。**

**证据：**KEYNOTE-024研究^[114]^是一项免疫检查点抑制剂帕博利珠单抗单药对比化疗一线治疗PD-L1高表达(TPS≥50%)的*EGFR*/*ALK*突变阴性的晚期NSCLC的全球多中心、随机对照Ⅲ期临床研究，研究中的年龄亚组分析显示，≥65岁人群中帕博利珠单抗OS获益与整体人群一致，显著优于化疗(HR=0.64, 95%CI: 0.42-0.98)。另一项针对KEYNOTE-024、KEYNOTE-042和KEYNOTE-010研究老年人群的汇总分析^[115]^显示，对于其中≥75岁、PD-L1高表达的NSCLC初治患者，帕博利珠单抗对比化疗显著改善OS(mOS：27.4个月 *vs* 7.7个月，HR=0.41，95%CI：0.23-0.73)。日本一项前瞻性、真实世界单臂研究^[116]^纳入了47例≥75岁、PD-L1高表达晚期NSCLC初治患者，帕博利珠单抗治疗ORR为53.1%，DCR为74.4%，mPFS为7.0个月，与KEYNOTE-024中整体人群获益基本一致。PePS2研究^[117]^是日本一项前瞻性、单臂、小样本Ⅱ期临床研究，旨在评价帕博利珠单抗治疗PS为2分的晚期NSCLC的疗效及安全性，患者中位年龄72岁，在PD-L1高表达的患者中持续临床获益率(DCR超过18周)为53%，显示PS为2分的老年患者选用帕博利珠单抗仍有可能获益。IMpower110研究^[118]^亚组分析提示，在≥65岁PD-L1高表达NSCLC中阿替利珠单抗对比化疗，有OS获益趋势，但未达到统计学意义。EMPOWER Lung-01研究^[119]^中的亚组数据分析看到，西米普利单抗对比化疗在≥65岁的PD-L1高表达的晚期NSCLC患者，无论是OS还是PFS都显著获益，但其中亚裔(*n*=77)接受西米普利单抗治疗相比化疗并不能降低死亡风险(HR=1.34)。

在二线及以上治疗中，多项临床研究^[120-122]^均显示ICIs单药在≥65岁人群显著获益。国外一项汇总分析纳入了KEYNOTE-010、OAK、POPLAR、CheckMate-057研究^[123]^，≥65岁和≥70岁的经治老年NSCLC患者中ICIs单药对比单药化疗OS均有获益，HR分别为0.66(95%CI: 0.57-0.76)、0.67(95%CI: 0.55-0.82)，≥75岁患者也有OS获益趋势(HR=0.81, 95%CI: 0.58-1.13)。CheckMate-171研究^[124]^是纳武利尤单抗治疗经治晚期肺鳞癌的Ⅱ期临床研究，≥70岁和≥75岁老年人群mOS分别为10.0个月、11.2个月，和整体人群10.0个月基本一致。CheckMate-153^[125]^是纳武利尤单抗治疗经治晚期NSCLC的Ⅲb期/Ⅳ期研究，主要基于社区进行，更接近于真实世界中的数据，≥70岁老年患者的mOS为10.3个月，2年OS率为25%，与整体人群的9.1个月、2年OS率26%基本相当。而在针对中国驱动基因阴性晚期NSCLC患者开展的二线免疫治疗CheckMate-078研究亚组分析中，≥65岁人群纳武利尤单抗对比化疗也有显著OS获益^[126]^。

**问题2：对于老年晚期NSCLC患者免疫联合治疗的疗效如何？**

**共识推荐：在老年晚期NSCLC免疫联合治疗中，ICIs联合化疗有临床获益。**

**证据：**目前免疫联合治疗模式有很多，有Ⅲ期临床研究数据支持的晚期NSCLC免疫联合治疗的模式包括免疫联合化疗(KEYNOTE-189、KEYNOTE-407、CameL、CameL-SQ、ORIENT-11、ORIENT-12、RATIONALE-307)、免疫联合抗血管药物治疗(IMpower150)、免疫双药联合治疗(CheckMate-227)及免疫双药联合化疗(CheckMate-9LA)等研究。

根据KEYNOTE-189研究^[127]^亚组数据，帕博利珠单抗联合化疗在≥65岁非鳞NSCLC中OS有显著获益(HR=0.64, 95%CI: 0.43-0.95)。KEYNOTE-407研究^[128]^≥65岁鳞癌亚组中，帕博利珠单抗联合化疗组虽然PFS有显著获益(HR=0.63, 95%CI: 0.47-0.84)，但OS没有达到显著差异。CameL-SQ研究^[129]^亚组数据结果，卡瑞利珠单抗联合化疗在≥65岁肺鳞癌中，PFS显著获益(HR=0.49, 95%CI: 0.33-0.71)，OS也有获益趋势。CameL研究^[130]^亚组分析中，卡瑞利珠单抗联合化疗在≥65岁非鳞NSCLC中，PFS有获益趋势。ORIENT-11是信迪利单抗联合化疗对比化疗用于一线治疗非鳞状NSCLC的随机、双盲、安慰剂对照Ⅲ期研究^[131]^，亚组分析提示，PFS和OS在 > 60岁晚期NSCLC中均显著获益。而根据ORIENT-12研究^[132]^已有的研究结果也显示，PFS在 > 60岁鳞癌人群中显著获益。2021年美国临床肿瘤学会(American Society of Clinical Oncology, ASCO)会议上报道了RATIONALE 307研究^[133]^亚组分析显示，≥65岁晚期肺鳞癌中替雷利珠单抗联合化疗组PFS有获益趋势。一项基于美国国家癌症数据库86, 173例患者分析^[134]^显示，≥75岁患者接受ICIs治疗的OS比未接受ICIs治疗的患者显著延长OS(HR=0.61, 95%CI: 0.58-0.64)。

IMpower150研究中阿替利珠单抗联合化疗及贝伐珠单抗在≥65人群中PFS有获益^[135, 136]^。CheckMate-9LA研究^[137]^中，纳武利尤单抗联合伊匹木单抗及2个周期诱导化疗在65岁-74岁NSCLC中OS显著优于单纯化疗。在CheckMate-227研究^[138]^中，纳武利尤单抗联合伊匹单抗对比化疗有生存获益趋势。目前对于≥75岁或者更高龄的老年患者，免疫联合抗血管治疗、双免联合治疗或者免疫联合化疗等治疗模式目前还没有足够证据支持，最佳免疫联合治疗模式对于高龄NSCLC患者未来需要更多相关研究探索。

**问题3：ICIs在老年NSCLC患者中安全性如何？**

**共识推荐：ICIs单药治疗在老年NSCLC中治疗相关AE发生率与整体人群类似。**

**证据：**ELDERS研究是一项针对老年患者ICIs安全性的前瞻性观察性队列研究，纳入了140例晚期NSCLC或恶性黑色素瘤患者进行ICIs单药(帕博利珠单抗、伊匹木单抗、纳武利尤单抗、阿替利珠单抗、度伐利尤单抗)治疗，对比≥70岁和 < 70岁患者的安全性，结果显示≥70岁人群中3级-5级免疫相关AE(immune-related AE, irAE)及所有级别irAE的发生率与 < 70岁人群基本一致，≥70岁和 < 70岁人群3级-5级irAEs发生率分别为18.6% *vs* 12.9%，所有级别irAEs的发生率分别为60% *vs* 51.4%^[17]^。一项汇总分析纳入了CheckMate-057、KEYNOTE-010、OAK、POPLAR研究^[123]^，≥75岁人群中3级-4级治疗相关AE(treatment-related AE, TRAE)的发生率低于 < 65岁和65岁-74岁年龄组，分别为23%、47%和49%；导致治疗终止的AE发生率相似，分别为5%、7%和7%。另一项包含KEYNOTE-010、KEYNOTE-024和KEYNOTE-042的汇总研究^[115]^结果显示，≥75岁人群和 < 75岁人群的TRAEs和irAEs的发生率相似，分别为68.5% *vs* 65.2%、24.8% *vs* 25.0%；≥75岁组最常见的irAEs包括甲状腺功能减退(8.7%)、肺炎(7.4%)、甲亢(5.4%)，≥3级irAEs的发生率为9.4%。CheckMate-153研究^[125]^显示，3级-5级TRAEs在≥70岁人群和总体人群中发生率一致，为6%。CheckMate-171研究^[124]^显示≥75岁人群和整体人群组任何级别TRAEs发生率分别为69%和57.3%，轻度腹泻在老年患者中更为常见，而其他AE类型和发生率则相似。2021年ASCO上也报道了一项对于2, 049例接受免疫治疗患者真实世界分析显示， > 75岁与≤75岁人群相比，irAEs发生率没有增加。目前老年人群中关于免疫联合化疗相对于单纯化疗AE发生率的研究数据报道不多。2021年ASCO上报道了对RATIONALE 307研究中≥65岁人群亚组分析显示，替雷利珠单抗联合化疗组对比单纯化疗组全级别和3级及以上TRAEs的发生率相似。综上，免疫治疗在老年NSCLC中治疗相关AE发生率与整体人群相似。

## 老年广泛期小细胞肺癌(small cell lung cancer, SCLC)的治疗

**问题1：老年广泛期SCLC患者治疗方案及剂量强度如何决策？**

**共识推荐：老年人群建议含铂双药联合方案，PS评分好的老年患者可与年轻人接受同样的剂量，而基础状态差、有危险因素的患者，首选低剂量含铂方案。**

**证据：**在老年人群中，鉴于器官生理功能储备下降和合并症的存在，广泛期SCLC的治疗更具挑战性。在随机试验^[139]^中表明，在PS评分0分-2分的老年患者中，低强度的治疗(如单药)疗效劣于含铂双药联合方案。多项临床试验^[140-143]^评估了低毒性治疗(单药治疗、单纯放疗和最佳支持治疗)在≥70岁老年SCLC患者中的作用；然而，这种低毒性的治疗可能会导致老年SCLC患者生存率下降。在日本开展的一个Ⅲ期临床研究^[140]^，纳入了220例广泛期SCLC，中位年龄74岁，其中92%的患者年龄 > 70岁(PS评分为0分-1分)。研究显示一线给予卡铂联合依托泊苷或剂量分割顺铂联合依托泊苷的两药联合方案对于老年广泛期SCLC可以获得更好的疗效，两组ORR均达到73%，MST分别为10.6个月和9.9个月。一项回顾性分析^[143]^展示了包含长春新碱、环磷酰胺和多柔比星的常规剂量化疗与低剂量方案化疗比较(单药化疗、计划剂量减量、仅放疗)的结果，其中32例老年SCLC患者进行了常规治疗，34例患者进行了低剂量方案的治疗。尽管常规治疗与较高的总生存率相关，但总体生存率无明显统计学差异，但常规治疗组的毒副反应显著增加。而Shepherd等^[142]^研究表明，接受常规化疗(环磷酰胺、多柔比星和长春新碱，或依托泊苷和顺铂)的 > 70岁老年SCLC患者比接受低剂量治疗(无单独放疗，少于3个化疗周期)OS明显延长。

对于PS评分≥3分及存在危险因素(如脑转移、肺部疾病史、存在合并症)的老年SCLC患者，尽管化疗毒性作用明显，但接受化疗的患者生存率明显提高^[144]^，应考虑风险-收益的平衡综合制定治疗方案。对于该类人群，低剂量的含铂化疗方案(如分次剂量化疗^[140]^或经剂量调整的含铂方案化疗^[145]^)与标准化疗相比生存获益并无明显差异，但毒性反应明显减少。

在临床实践中，对于PS评分高、基础状态差的老年患者，口服单药依托泊苷化疗也是一种治疗选择。口服依托泊苷在提高癌症患者的QOL和经济效益方面具有一定优势。有一项研究^[146]^纳入了1, 264例转移性SCLC患者，最高年龄达91岁，据统计分析，口服与静脉输注依托泊苷对比OS无明显统计学差异。一项关于晚期SCLC患者诱导化疗后口服依托泊苷维持治疗对比不再治疗的Ⅲ期试验^[147]^表明，PFS有明显改善，但OS没有明显延长。与之相反，一项Ⅲ期随机临床试验^[148]^对比了静脉注射伊立替康联合卡铂对比口服依托泊苷联合卡铂，在209例转移性SCLC患者中包含35%年龄≥70岁的患者及47% PS评分在2分-4分的患者。研究表明，静脉注射伊立替康组在mOS(8.5个月 *vs* 7.1个月，*P*=0.02)和1年生存率(34% *vs* 24%)方面都明显优于口服依托泊苷，但是伊立替康组发生3级-4级腹泻更常见。然而，由于毒性反应明显，在1/3的患者中(包括PS评分为3分-4分、年龄≥70岁)使用了减量的方案，因此该临床研究有其局限性。口服依托泊苷不失为老年SCLC患者的一种选择，而且与静脉注射不同，口服依托泊苷的药代动力学影响因素较多，不同患者的生物变异性很大，骨髓抑制是口服依托泊苷的主要毒性反应。相关研究表明，与同等药物暴露量的年轻患者相比，老年患者的毒性更严重^[149]^，因此口服药物期间需要密切观察药物的毒副反应^[150]^。

**问题2：老年广泛期SCLC患者如何选择卡铂和顺铂？**

**共识推荐：在老年患者中接受卡铂或者顺铂化疗后的生存无明显差异，治疗药物选择主要取决于患者年龄、PS评分和血液学毒性因素。**

**证据：**多项随机对照及回顾性研究结果表明，在老年患者中，包括卡铂或者顺铂的联合化疗方案对于患者的PFS/OS无显著差异。一项*meta*分析^[151]^系统性地回顾了几项研究共663例SCLC患者的临床预后数据，对比了依托泊苷联合卡铂或者顺铂的疗效差异，发现使用卡铂或者顺铂对于OS无明显差异(9.6个月 *vs* 9.4个月，*P*=0.37)。而对各个亚组进行探索性分析后，当考虑到患者如果相对年轻(< 70岁)、PS评分和血液学毒性因素时，顺铂可能是首选。据此，广泛期SCLC一线化疗方案可以考虑卡铂替代顺铂，而对于特定患者，如相对年轻和考虑血液毒性AE，可考虑首选顺铂。

日本的一项研究^[140]^针对年龄 > 70岁、PS评分在0分-3分的老年及基础状态较差的220例广泛期SCLC患者进行评估，分别接受依托泊苷联合卡铂(EC)或者顺铂(EP)方案治疗4个周期。结果发现ORR(73% *vs* 73%)和中位OS(10.6个月 *vs* 9.9个月，*P*=0.54)没有明显差异；3级-4级骨髓抑制的发生率也未见明显差异。尽管EP方案仍被认为是老年或低风险广泛期SCLC患者的标准治疗方法，但考虑到风险-收益的平衡，EC方案可以作为这一人群的替代方案。

HATFIELD等^[152]^通过对美国SEER数据库中1995年-2009年的超过69岁的831例接受EP方案和2, 846例接受EC方案的广泛期SCLC患者进行2:1区间配对分析，共纳入778例EP方案和1, 502例EC方案患者。发现两组之间生存接近，分别为35.7周和35.9周，6个月生存率分别为35%和34%，只是EC方案的患者治疗后住院比例更低，分别为80%和86% (*P* < 0.001)。可见对于老年患者，卡铂带来的毒性反应较顺铂低，耐受性更佳。

因此，对于年龄 > 70岁、PS评分在0分-2分、基础状态较好、无特殊合并症、可以耐受化疗的老年SCLC患者，使用卡铂和顺铂的生存期无明显差异，如果考虑到治疗相关AE，卡铂较顺铂的耐受性更佳。

**问题3：老年广泛期SCLC患者二线治疗如何选择？**

**共识推荐：对于一线治疗后停药时间间隔长(≥90 d)的患者，可考虑一线方案再挑战或其他二线治疗药物。**

**证据：**法国的一项随机对照研究^[153]^纳入了164例在一线依托泊苷联合铂类化疗后≥90 d进展SCLC患者，分别使用拓扑替康和EC方案。结果显示EC方案获得更好的生存和更高的缓解率，一线治疗后≥180 d进展患者获益最佳，≥90 d且 < 180 d进展患者或≥70岁的患者亦有更优趋势。提示对于敏感复发的SCLC患者，二线可选择卡铂联合依托泊苷的再挑战。日本的一项回顾性研究^[154]^分析显示，在接受二线化疗的复发老年SCLC患者中，PS评分良好、较早接受一线治疗且一线治疗与二线治疗的时间间隔越长的患者，其OS获益可能会明显。

2020年，鲁比卡丁被FDA获批上市，并被NCCN指南纳入作为含铂化疗复发后转移性SCLC二线治疗可选方案。这是近20年来SCLC在化疗中的首次突破进展。Ⅰ期临床研究^[155]^结果显示，鲁比卡丁二线治疗SCLC的ORR达到35.2%，中位持续缓解时间(median duration of response, mDOR)为5.3个月。根据Ⅱ期临床试验^[156]^结果，应用鲁比卡丁方案二线治疗的SCLC患者，在随访时间达到17.1个月时，ORR为35.2%，是一种安全有效的治疗方法。在Ⅰ期研究^[155]^的105例患者中，≥75岁的SCLC患者占9%，PS评分为0分-2分，主要的AE为血液学毒性，但该研究未对高龄患者单独分析。目前鲁比卡丁未在中国上市，也还未有相关数据证明鲁比卡丁在老年SCLC患者中的疗效及毒性反应，但是鲁比卡丁不失为一种新的治疗方案选择。

**问题4：老年SCLC患者免疫检查点抑制剂治疗是否能带来生存获益？**

**共识推荐：ICIs联合化疗可以延长老年广泛期SCLC患者生存时间，但应关注老年人群的基础状态及可能因治疗导致的AE。**

**证据：**根据IMpower133^[157]^和CASPIAN^[158]^的研究数据，ICIs联合化疗成为广泛期SCLC患者的一线治疗选择。IMpower133^[157]^研究中亚组分析显示在≥65岁患者阿替利珠单抗联合化疗较单纯化疗明显获益，而 < 65岁患者获益不明显。在CASPIAN^[158]^研究中， < 65岁和≥65岁的人群对于度伐利尤单抗联合化疗的方案均获益。根据ASCO 2021的报道^[159]^，与IMpower133研究相比，真实世界中的患者基线特征(年龄、脑转移、PS评分)更差，中位年龄达到68岁，最高年龄达88岁，但发现该描述性分析中的真实世界中位开始用药至治疗终止时间(time to treatment discontinuation, TTD)与临床研究中的中位治疗持续时间一致。

在真实世界数据研究^[160]^中，高达1/3的SCLC患者的PS评分为2分，15%的患者PS评分为3分-4分。然而，在老年SCLC患者中，高达60%的患者PS评分为2分，这与较差的生存率有关^[161]^。正在进行的Ⅱ期SPACE试验(NCT04221529)和Ⅲ期MAURIS试验(NCT04028050)招募PS评分 > 2分的SCLC患者，可能有助于阐明这些患者是否从化疗联合ICIs治疗中获益。

一项*meta*分析^[162]^纳入了包括IMpower133、CASPIAN、KEYNOTE-604在内的2, 775例患者，结果显示在年龄 < 65岁和≥65岁、PS评分为0分-1分的亚组中，免疫联合化疗的队列都明显获益。分析表明免疫联合化疗的优势在老年SCLC患者中也有体现，但与单纯化疗相比，联合治疗发生的3级-4级AE(如皮疹、输注相关AE、结肠炎等)概率更高。因此老年广泛期SCLC患者可以从免疫联合化疗中获益，但是要关注患者的基础状态以及特别关注在治疗过程中的相关AE。

## 附件

**量表 1:** 癌症及衰老研究组(CARG)化疗风险评估量表

内容		评分
年龄	≥72岁	2
	< 72岁	0
肿瘤类型	消化道肿瘤	2
	其他肿瘤	0
计划化疗药用量	标准剂量	2
	减量	0
化疗药数量	多药方案	2
	单药方案	0
血红蛋白	男性 < 11 g/dL，女性 < 10 g/dL	3
	男性 > 11 g/dL，女性 > 10 g/dL	0
肌酐清除率	< 34 mL/min	3
	≥34 mL/min	0
听力	差	2
	好	0
近6个月内摔倒次数	≥1	3
	无	0
服药是否需要帮助	是	1
	否	0
行动是否受限	是	2
	否	0
近4周社会活动是否受限	是	1
	否	0
低危：0分-5分，中危险：6分-9分，高危：≥10分。		

**量表 2:** 老年化疗风险评估量表(CRASH)

内容		评分			结果	
		0	1	2		
血液学(H)评分	舒张压	≤72	> 72		低危：0分-1分； 中低危：2分-3分； 中高危：4分-5分； 高危：6分-8分	综合评分：(化疗方案只加一次) 低危：0分-3分； 中低危：4分-6分； 中高危：7分-9分； 高危：≥10分
	IADL*(见附表 1)	26-29	10-25			
	LDH	0-459		> 459		
	化疗方案(见附表 2)					
非血液学(NH)评分	ECOG PS	0	1-2	3-4	低危：0分-2分； 中低危：3分-4分； 中高危：5分-6分； 高危：7分-8分	
	MMSE(见附表 3)	30		< 30		
	MNA(见附表 4)	28-30		< 28		
	化疗方案(见附表 2)					
IADL：工具性日常生活活动能力量表；LDH：乳酸脱氢酶；MMSE：Folstein简易智能量表；MNA：微型营养评估量表。 *：Extermann团队提出CRASH量表时采用的IADL量表为Lawton 9项IADL量表，本文所引用量表为目前临床常用Lawton 8项IADL量表。						

**附表 1:** 工具性日常生活活动能力量表(IADL)

问题	项目	评分	得分
使用电话的能力	主动操作电话查找和表盘数字	1	
	拨打几个熟悉的数字	1	
	接电话但不打电话	1	
	不使用电话	0	
购物	独立地购买所有必需品	1	
	独立地购买小型物品	0	
	需要别人陪同购物	0	
	完全不能购物	0	
准备食物	独立计划、准备和制作足够的食物	1	
	能按清单准备足够食物	0	
	加热、制作和准备食物，但不保持足够的饮食	0	
	需要别人帮助提供食物和用餐	0	
家务	独立做家务或遇上重体力家务偶尔需要请家政帮忙	1	
	能做简单的家务(比如洗完铺床)	1	
	能做简单的家务但不能保持家里清洁	1	
	需要别人帮助才能完成所有家务	1	
	不参与任何家务	0	
洗衣	能独立洗衣服	1	
	能洗小件物品(如袜子)	1	
	所有衣服必须由别人洗	0	
出行方式	能独立搭乘公共交通工具或自己开车	1	
	能自行安排乘坐出租车出行，但不能乘坐其他公共交通工具	1	
	在别人陪同下能乘坐公共交通工具	1	
	仅能在别人陪同下乘坐出租车或汽车	0	
	根本不能出行	0	
服用药物	能独立准时按剂量吃药	1	
	在药物预先准备好剂量的情况下能服用	0	
	不自行分配和服用自己的药物	0	
处理财务能力	独立处理自己的财务(预算、写支票、支付租金、去银行)，管理个人收入	1	
	能处理购物，但去银行或重要购买时需要帮助	1	
	不能处理金钱	0	
每个问题选择一个选项，总计0分-8分。			

**附表 2:** 化疗方案评分^a^

0	1	2
卡培他滨2 g	卡培他滨2.5 g	5-FU/LV
顺铂/培美曲塞	卡铂/吉西他滨AUC 4-6/1 g d1, d8	5-FU/LV+贝伐珠单抗
达卡巴嗪	卡铂/培美曲塞	CAF
多西他赛*qw*	卡铂/紫杉醇*qw*	卡铂/多西他赛*q3w*
FOLFIRI	顺铂/吉西他滨d1, d8	CHOP
吉西他滨1 g 3/4 w	ECF	顺铂/多西他赛75/75 mg
吉西他滨1.25 g 3/4 w	氟达拉滨	顺铂/依托泊苷
紫杉醇*qw*	FOLFOX 85 mg	顺铂/吉西他滨d1, d8, d15
培美曲塞	吉西他滨7/8 w→3/4 w	顺铂/紫杉醇135 mg *q3w*
	吉西他滨/伊立替康	CMF
	聚乙二醇化多柔比星50 mg *q4w*	多柔比星*q3w*
	拓扑替康*qw*	FOLFOX 100 mg-130 mg
	XELOX	吉西他滨/培美曲塞d8
		伊立替康*q3w*
		紫杉醇*q3w*
		多西他赛*q3w*
		拓扑替康*qm*
5-FU：5-氟尿嘧啶；AUC：曲线下浓度；CAF：环磷酰胺，多柔比星，5-氟尿嘧啶；CHOP：环磷酰胺，多柔比星，长春新碱，强的松；CMF：环磷酰胺，甲氨蝶呤，5-氟尿嘧啶；ECF：表柔比星，顺铂，5-氟尿嘧啶；FOLFIRI：伊立替康，甲酰四氢叶酸，5-氟尿嘧啶；FOLFOX：四氢叶酸，5-氟尿嘧啶，奥沙利铂；LV：甲酰四氢叶酸；q3w：每3周；q4w：每4周；XELOX：卡培他滨，奥沙利铂；3/4w：每用3周休1周；7/8w→3/4w：首先连用7周休1周，之后每用3周休1周。 ^a^如果不特殊说明，剂量均按每平方米计算。如果未标注剂量，则表明该方案的常用剂量均适用。		

**附表 3:** 简易智能量表(MMSE)

项目		得分备注
定向力(10分)	今年是哪一年？	每个问题记一分
	现在是什么季节？	
	现在是几月份？	
	今天是几号？	
	今天是星期几？	
	您住在哪个省？	
	您住在哪个县(区)？	
	您住在哪个乡(街道)？	
	咱们现在在哪个医院？	
	咱们现在在第几层楼？	
记忆力(3分)	告诉您三种东西，我说完后，请你重复一遍并记住，待会还会问您	各1分，共3分
注意力和计算力(5分)	100-7=？连续减5次(93、86、79、72、65)	各1分，共5分，若错了，但下一个答案正确，只记一次错误
回忆能力(3分)	现在请您说出我刚才告诉您让您记住的那些东西	各1分，共3分
语言能力(9分)	命名能力(出示钢笔和手表)	各1分，共2分
	复述能力(四十四只石狮子)	1分
	阅读能力(“闭上你的眼睛”念这句话并按要求做)	1分
	三步命令：我给您一张纸，请按照我说的去做：用右手拿着这张纸，用两只手将它对折起来，放在您的左腿上	每个工作1分，共3分
	书写能力：要求受试者自己写一句完整的句子	1分
	结构能力：(出示图案)请您按照上面图案画下来	1分

**附表 4:** 微型营养评估(MNA)量表

项目			评分
体格检查	BMI(kg/m^2^)	BMI < 19	0
		19≤BMI < 21	1
		21≤BMI < 23	2
		BMI≥23	3
	上臂肌肉周径(MAC)(cm)	MAC < 21	0
		21≤MAC≤22	0.5
		MAC > 22	1
	腓肠肌周径(CC)(cm)	CC < 31	0
		CC≥31	1
	3个月内体重减轻	> 3 kg	0
		未知	1
		1 kg -3 kg	2
		无体重减轻	3
一般情况	独自居住	否	0
		是	1
	每日药物超过3种	是	0
		否	1
	3个月内心理压力或急性病	是	0
		否	2
	活动能力	需要轮椅或卧床	0
		不需卧床，无外出	1
		可外出	2
	神经精神问题	严重痴呆或抑郁	0
		中度痴呆	1
		无	2
	压疮或皮肤溃疡	有	0
		无	1
饮食评估	每日摄入正餐数量	1餐	0
		2餐	1
		3餐	2
	蛋白质摄入情况	每日摄入至少一次牛奶、奶酪、酸奶等	0分：无或仅1项； 0.5分：2项； 1分：3项
		每周摄入至少两次豆类、鸡蛋等	
		每日摄入鱼、肉、禽类等	
	每日蔬果摄入	未达2次	0
		2次及以上	1
	3个月内有无食欲减退导致的饭量减少	严重食欲减退	0
		中度食欲减退	1
		无食欲减退	2
	每日液体摄入量	小于3杯	0
		3杯-5杯	0.5
		多于5杯	1
自评	是否自认为存在营养不良	存在	0
		未知	1
		无	2
	和同龄人相比的健康状况	差于同龄人	0
		未知	0.5
		相同	1
		优于同龄人	2
MNA评分结果： ≥24分：营养良好 17分-23.5分：存在营养风险 < 17分：营养不良			

**Table:** 参与本共识的专家组成员(按姓氏汉语拼音排名)

	姓名	工作单位
顾问专家	陈军	天津市肺癌研究所，天津医科大学总医院
	程刚	北京医院，国家老年医学中心，中国医学科学院老年医学研究院
	程颖	吉林省肿瘤医院
	胡毅	中国人民解放军总医院肿瘤医学部
	王洁	国家癌症中心，国家肿瘤临床医学研究中心，中国医学科学院北京协和医学院肿瘤医院
	周彩存	上海市肺科医院
执笔专家	黄鼎智	天津医科大学肿瘤医院，国家恶性肿瘤临床医学研究中心，天津市恶性肿瘤临床医学研究中心
	李琳	北京医院，国家老年医学中心，中国医学科学院老年医学研究院
	李旭	北京医院，国家老年医学中心，中国医学科学院老年医学研究院
	刘东颖	天津医科大学肿瘤医院，国家恶性肿瘤临床医学研究中心，天津市恶性肿瘤临床医学研究中心
	聂鑫	北京医院，国家老年医学中心，中国医学科学院老年医学研究院
	唐敏	北京医院，国家老年医学中心，中国医学科学院老年医学研究院
	万蕊	国家癌症中心，国家肿瘤临床医学研究中心，中国医学科学院北京协和医学院肿瘤医院
	王志杰	国家癌症中心，国家肿瘤临床医学研究中心，中国医学科学院北京协和医学院肿瘤医院
	邬麟	湖南省肿瘤医院，中南大学湘雅医学院附属肿瘤医院
	徐燕	中国医学科学院，北京协和医院
参加撰写	高佳仪	北京医院，国家老年医学中心，中国医学科学院老年医学研究院
	李梦洁	天津医科大学肿瘤医院，国家恶性肿瘤临床医学研究中心，天津市恶性肿瘤临床医学研究中心
	李昕	天津市肺癌研究所，天津医科大学总医院
	蒲兴祥	湖南省肿瘤医院，中南大学湘雅医学院附属肿瘤医院
	王婧雅	天津医科大学肿瘤医院，国家恶性肿瘤临床医学研究中心，天津市恶性肿瘤临床医学研究中心
	王婧怡	湖南省肿瘤医院，中南大学湘雅医学院附属肿瘤医院
	徐嵩	天津市肺癌研究所，天津医科大学总医院
	杨帆	北京医院，国家老年医学中心，中国医学科学院老年医学研究院
	杨广建	国家癌症中心，国家肿瘤临床医学研究中心，中国医学科学院北京协和医学院肿瘤医院
	袁月	北京医院，国家老年医学中心，中国医学科学院老年医学研究院
